# Influence of long-term thermal stress on the  *in vitro* maturation on embryo development and Heat Shock Protein abundance in zebu cattle

**DOI:** 10.1590/1984-3143-AR2019-0085

**Published:** 2020-08-27

**Authors:** Ralf Pöhland, Mirela Brochado Souza-Cácares, Tirtha Kumar Datta, Jens Vanselow, Maria Isabel Mello Martins, Wilian Aparecido Leite da Silva, Christopher Junior Tavares Cardoso, Fabiana de Andrade Melo-Sterza

**Affiliations:** 1 Institute of Reproductive Biology, Leibniz Institute for Farm Animal Biology, Dummerstorf, Germany; 2 Programa de Pós-graduação em Ciência Animal, Universidade Estadual de Londrina, Londrina, PR, Brasil; 3 National Dairy Research Institute, Animal Biotechnology Centre, Karnal, Haryana, India; 4 Programa de Pós-graduação em Ciência Animal, Universidade Federal de Mato Grosso do Sul, Campo Grande, MS, Brasil; 5 Programa de Pós-graduação em Ciências Veterinárias, Universidade Federal de Mato Grosso do Sul, Campo Grande, MS, Brasil; 6 Programa de Pós-graduação em Zootecnia, Universidade Estadual de Mato Grosso do Sul, Aquidauana, MS, Brasil

**Keywords:** oocytes, HSP70, HSP90, bovine

## Abstract

The objective of this study was to investigate the influence of long-term temperature stress during the *in vitro* maturation (IVM) of oocytes on the *in vitro* embryo production (IVP) and the abundance of HSP70 and HSP90 in zebu cattle. Viable cumulus-oocyte complexes (COCs) were incubated for 24 h at 37 °C, 38.5 °C, or 40 °C for the low-, physiological, and high-temperature stress treatments, respectively. Thereafter, they were subjected to *in vitro* fertilization and culture. Temperature did not affect the polar body extrusion. However, IVP was adversely affected when IVM took place at 37 °C and 40 °C. The highest abundance of HSP70 was observed in cumulus cells after maturation of COCs at 40 °C. In contrast, HSP70 was more abundant in oocytes at both 37 °C and 40 °C; however, at 40 °C, the difference to the control group (38.5 °C) was not significant. In contrast, the highest abundance of HSP90 was observed in oocytes and cumulus cells at 37 °C. It appears that HSP70 and HSP90 respond to cold and heat stress in different ways. In conclusion, moderately high (40 °C) and low (37 °C) thermal stress for 24 h during IVM is detrimental to the developmental competence of oocyte and is accompanied by changes in the abundances of HSP70 and HSP90, especially in cumulus cells.

## Introduction

The reduced performance of cows subjected to high temperatures is well known. Heat stress (HS) is associated with early embryonic mortality (Edwards et al., 2005), decreased the conception rate (García-Ispierto et al., 2007), changes in steroid hormone levels (Wolfenson et al., 2000; Jasnic et al., 2010) and follicular wave patterns, and physiological and molecular alterations in oocytes (Zeron et al., 2001; Torres-Junior et al., 2008; Ferreira et al., 2011). Nevertheless, zebu breeds are better able to regulate body temperature and experience less severe impairment of productive and reproductive functions in response to heat stress (reviewed by Hansen, 2004). However, it has been demonstrated that a high temperature-humidity index during the tropical summer, around artificial insemination time, reduced the viability of embryos obtained from superovulated zebu females (Macedo et al., 2012).

The possibility of low-temperature stresses affecting tropical-adapted breeds was discussed by Souza-Cácares et al. (2019), who showed that the oocyte quality, obtained by ovum pick-up from Pantaneira heifers (a tropical-adapted breed), was lower and the abundance of Heat Shock Protein (HSP) 70 was higher when Black globe-humidity was lower than 80. These results encouraged us to evaluate whether heat and cold stress have the same effects on competence and HSP expression in zebu-derived oocytes.

HSPs are chaperones proteins that protect other proteins against acute heat at the cellular level (Maloyan et al., 1999; Daugaard et al., 2007). The HSPs are divided into groups based on their molecular size (Beachy et al., 2007; Romanucci et al., 2006) and they protect the cell by minimizing the accumulation of denatured or abnormal proteins in the cell (reviewed by Hassan et al., 2019).

The HSP70 and HSP90 chaperone machines participate in many cellular processes under physiological and different stress conditions. HSP90 acts downstream of HSP70 to improve protein folding and to optimize the maturation of key regulatory proteins (reviewed by Morán-Luengo et al., 2018).

Heat Stress (HS) has adverse effects on oocyte maturation, both *in vivo* (Gendelman and Roth, 2012) and *in vitro* (Nabenishi et al., 2012a). For example, HS can cause an increase in the rates of apoptosis (Roth and Hansen, 2004) and reactive oxygen species (ROS) (Nabenishi et al., 2012a), as well as a decrease in the proportion of oocytes that reach metaphase II (MII) *in vitro* (Nabenishi et al., 2012a; Maya-Soriano et al., 2013). In addition, HS can accelerate germinal vesicle breakdown (Hooper et al., 2015). However, there appears to be a compensatory effect, with the blockade of these oocytes in metaphase I, anaphase I, and telophase I consequently reducing the proportion of oocytes reaching MII (Roth and Hansen, 2005). In contrast with HS, the consequences of oocyte and embryo development in low-temperature conditions at the molecular level are unknown.

Numerous studies have assessed the influence of different temperatures on cell culture. However, in most studies, temperature increases were maintained for only a few hours and/or very high temperatures were used (Roth and Hansen, 2005; Nabenishi et al., 2012a, b; Rispoli et al., 2013). We performed IVM under mild heat stress conditions (40 °C), considering that the temperature at which the follicle/ cumulus-oocyte complex (COC) is subjected to *in vivo* is equal to or slightly higher than the body temperature of the animal (El-Sheikh Ali et al., 2013), and focused on chronic stress.

Thus, the present study was performed to investigate the effects of moderately high (40 °C) and low (37 °C) temperatures during the entire *in vitro* maturation (IVM) of zebu COCs on oocyte and embryo development, as well as on the abundance of the HSP70 and HSP90 proteins.

## Material and methods

Unless otherwise mentioned, the reagents used in this experiment were purchased from Sigma (St. Louis, MO, USA).

### *in vitro* embryo production

The ovaries were collected from a local slaughterhouse and transported in saline solution with penicillin/streptomycin (37 °C) to the laboratory within 2 h. Ovaries were collected only from cows with phenotypic zebu characteristics: white or gray color and showing humps above their shoulders.

Follicles (2-8 mm) were aspirated with syringes and 18G needles. The follicular fluid was kept in a water bath (37 °C) until sedimentation and pellet formation was completed. The pellet contents were screened in medium containing 10% fetal calf serum (FCS) and Dulbecco’s phosphate buffered saline (DPBS). The COCs were then selected for IVM according to the number of cumulus cell layers and cytoplasm homogeneity (Stojkovic et al., 2001).

The selected COCs were matured for 24 h in drops of 100 μL of the maturation medium (TCM199 with Earl’s salts, FCS 5% v/v, 0.5 ng/mL estradiol, 0.01 mIE/mL LH, 200 mM L-glutamine, 0.01 mg/mL streptomycin, and 10 U/mL penicillin), covered with mineral oil in an incubator (5% CO_2_, 5% O_2_, 90% N_2_; EVE-WTA, Brazil) at maximum humidity. The obtained COCs were divided into three groups according to the incubator temperature (37 °C, 38.5 °C [control], and 40 °C).

After maturation for 24 h, the COCs (n = 911; 3 replicates) were partially denuded (successive pipetting) and evaluated under a stereomicroscope to observe the extrusion of the first polar body (PB). We calculated the PB extrusion rate by considering the number of oocytes with PB compared with the total number of COCs subjected to IVM at each temperature.

After the maturation period, groups of 25 oocytes (n = 449) were transferred to 100 μL of Fert-Talp supplemented with 5 mg/mL BSA, 0.2 mM pyruvate, 30 μg/mL heparin, 18 μM penicillamine, 10 μM hypotaurine, 1.8 μM epinephrine, 100 μg/mL streptomycin sulfate, and 100 IU/mL penicillin, and covered with mineral oil. The oocytes were subjected to *in vitro* fertilization (IVF) with frozen semen from a single Nelore bull with proven fertility. Thawed sperms were washed in a discontinuous 45%/90% Percoll gradient (Parrish et al., 1995), and the concentration was adjusted to 1 × 10^6^ sperm/mL. Sperm and COCs were co-incubated under the same conditions as used for IVM for 18-22 h. The fertilization day was considered day (d) zero.

Putative zygotes were stripped of cumulus cells and spermatozoa by gentle pipetting into embryo culture medium (SOFaa, 250 mg/mL amikacin, and 5% FCS). *In vitro* culture (IVC) was performed in the same medium for 7 d.

IVF and IVC were performed at 38.5 °C, under high humidity and low oxygen tension (5% CO_2_, 5% O_2_, 90% N_2_ - EVE-WTA, Brazil).

The cleavage and blastocyst rates were evaluated at 72 h (d 3) and 7 d after fertilization, respectively.

### Electrophoresis and western blotting

Only oocytes with PB were prepared for western blotting analysis (n=180).

Western blotting analysis was performed as described by Poehland et al. (2008). Matured COCs were separated mechanically (frequent pipetting with a 100 µL automatic pipette) until the denuded oocytes were obtained. The denuded oocytes were collected and washed three times with PBS. Twenty oocytes per group (performed for three replicates) were transferred with a minimum volume of PBS (smallest possible, not more than 5 µL) into tubes containing 1 mL of 2× SDS sample buffer. The cumulus cells belonging to the respective oocytes were collected and washed three times with PBS. The precipitates of the last washing steps were resuspended in 5 mL of 2× SDS sample buffer in tubes. All samples were stored at -20 °C until western blotting.

SDS-PAGE 10% and 12.5% (w/w) gels (acrylamide to bisacrylamide ratio, 29.7:0.3) were used as described by Laemmli (1970). After rapid thawing, the probes were lysed and denatured for 2 min at 95 °C, and then immediately loaded onto the gel. Electrophoresis was performed (30 mA, 1-2 h) using a Mighty Small SE 250 system (Hoefer, Amersham Biosciences, Freiburg, Germany). The proteins were transferred to polyvinylidene difluoride (PVDF) membranes using a semidry electroblotting apparatus (OWL/peQLab, Erlangen, Germany, 1 mA/cm^2^, 1 h).

The samples were probed for HSP70 and HSP90 with antibodies diluted in 5% bovine serum albumin (BSA) in Tween Tris-buffered saline (TTBS: pH 7.4, 0.1% (v/v) Tween (see Table 1). The membranes were blocked in 0.5% (w/v) fat-free dry milk in TTBS using the SNAP system. Thereafter, the membranes were washed three times with TTBS (10 min each). The incubation for the primary antibodies was performed for 1 h at room temperature, and the antibodies were diluted as shown in Table 1. The blots were incubated with secondary antibodies (donkey anti-rabbit horseradish peroxidase; see Table 1) for 2 h at room temperature. The protein bands were visualized on X-ray films (Kodak, Rochester, NY) using an enhanced chemiluminescence (ECL) kit ECL+ and ECL (Amersham Pharmacia Biotech, Freiburg, Germany).

**Table 1:** Antibodies and dilutions used for western blotting (additional information: CST: Cell Signaling Technology).

Target	Antibody ID	Description	Dilution
HSP70	CST #4872	primary rabbit anti-human HSP70 (polyclonal)	1:700
HSP90	CST #4874	primary rabbit anti-human HSP90 (polyclonal)	1:700
2.Ab	CST #7074	Anti-rabbit IgG, HRP-linked Antibody	1:2500

### Statistical analysis

The experimental design was completely randomized. R software (open source) was used to for data analysis. First, the normality of the data was verified using the Shapiro-Wilk test. Then, for parametric data (HSP70 and HSP90), ANOVA was performed. When a significant difference was found, Tukey’s test was performed, at 5% probability. For non-parametric data (PB extrusion rate, cleavage rate, and blastocyst rate), the Kruskal-Wallis test was performed.

### Ethical standards

Not applicable.

## Results

We estimated the nuclear maturation success of the oocytes via observation of the first polar body extrusion; this was similar in all IVM temperature conditions (P=0.85; Table 2). However, the cleavage (P=0.02) and blastocyst (P=0.001) rates (Table 2) were lower when COCs were matured at 37 °C and 40 °C than those matured at 38.5 °C.

**Table 2:** Polar body extrusion rate, cleavage rate, and blastocyst rate after *in vitro* maturation at different temperatures (37 °C, 38.5 °C, and 40 °C).

Temperature (°C)	Maturation rate (%)	Cleavage rate (%)	Blastocyst rate (%)
37	75 (212/281)	35 (55/154)^b^	14 (23/154)^b^
38.5	80 (271/336)	76 (116/152)^a^	32 (50/152)^a^
40	74 (219/294)	26 (38/143)^b^	11 (16/143)^b^
P-value	0.8563	0.025	0.001

^ab^Within a column, means with different superscripts are different.

**Figure 1:**
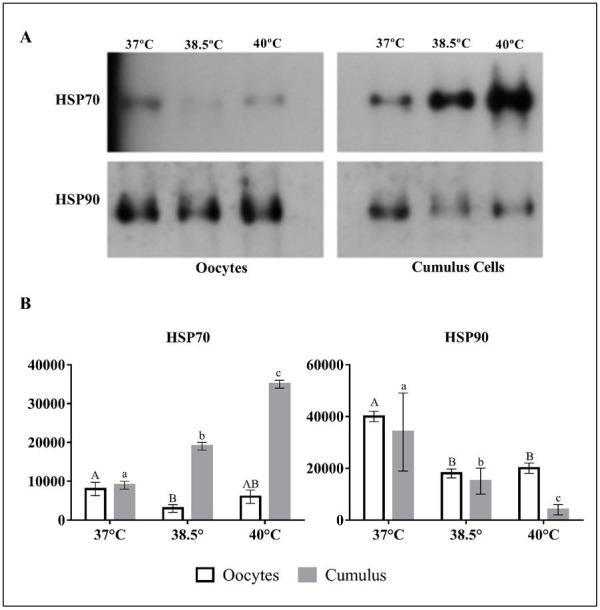
HSP70 and HSP90 abundance in zebu oocytes and cumulus cells after *in vitro* maturation at 37 °C, 38.5 °C, and 40 °C. (A) Examples of western blotting images (exposure time, 2 min); (B) Quantitative evaluation is represented by diagrams (mean and SD; three biological repeats). Different uppercase and lowercase letters indicate a significant difference (P < 0.05).

In cumulus cells, a temperature-dependent increase was observed for HSP70 (P< 0.05). In contrast, in oocytes, the abundance was higher in the control group at 38.5 °C than at 37 °C and 40 °C; however, at 40 °C, the difference from COCs matured at 38.5 °C was not significant (Figure 1). HSP90 showed the highest levels at 37 °C and a significant decrease as the maturation temperature increased (P< 0.05). The oocytes showed the highest levels of HSP90 when matured at 37 °C (P< 0.05; Figure 1), but the levels were similar between COCs matured at 38.5 °C and 40 °C.

## Discussion

It is known that zebu breeds are more tolerant to heat (Hansen, 2004). However, there is a limit to this, as heat stress at different temperatures and durations during the IVM of zebu COCs impairs *in vitro* embryo development (Lima et al., 2017; reviewed by Paula-Lopes et al., 2013). However, to the best of our knowledge, no study has demonstrated the effects of low-temperature stress during the IVM of bovine oocytes.

Maya-Soriano et al. (2013) collected bovine COCs during warm and cold periods of the year and subjected them to IVM under heat stress. They showed that COCs obtained in colder periods were less tolerant than those obtained in warmer periods. In the present study, zebu COCs were collected during a winter with moderate temperatures and were subjected to moderately high (40 °C) or low (37 °C) temperature during the entire IVM. In both situations, embryo development was negatively affected, indicating similar stresses.

In our study, no differences in the PB extrusion rate were observed between the thermally stressed (37 °C and 40 °C) and non-stressed COCs. The extrusion of the first polar body can indicate the success of nuclear maturation (Halvaei et al., 2011), but does not provide information about cytoplasmic maturity. To complete maturation, many intracellular events are necessary in the oocyte, including the redistribution of cytoplasmic organelles, storage of messenger RNA (mRNA), transcription factors, and protein synthesis (Reader et al., 2017; Conti and Franciosi, 2018). The success of embryonic development depends on the adequate synchronicity of these events (Conti and Franciosi, 2018). Probably, the thermal stress to which COCs were submitted in the present study affected only cytoplasmic and/or molecular processes. Hooper et al. (2015) showed that COCs subjected to HS (41 °C) in the first 12 h of IVM reached metaphase II (MII) at similar rates to non-stressed COCs (91.6% vs. 91.1%); however, germinal vesicle breakdown (GVBD) in heat-stressed oocytes was rushed, as the majority reached GVBD in the first 6 h of maturation. The same authors showed a higher amount of ATP in HS oocytes after maturation for 24 h, which aligns with aging. In addition, cytoskeletal changes and apoptosis were observed in oocytes after HS during the first 12 h of maturation, which might trigger the impairment of further embryo development (Roth and Hansen, 2005). In contrast to Hooper et al. (2015) and in contrast to our results, Roth and Hansen (2005) found a significant reduction in the proportion of oocytes in the MII phase after IVM for 12 h at 40 °C or 41 °C followed by maturation for 10 h at 38.5 °C. We can only speculate about the reasons for these different results. However, it is conceivable that differences in IVM methods and influences of different animal breeds are responsible for this. As previously mentioned, nuclear maturation is not a complete marker of oocyte maturation. Ultimately, only developmental competence after fertilization is relevant.

In somatic cells, different studies have shown that the cellular physiological effects of cold stress are similar to those seen in heat-stressed cells, such as an increase in the denaturation of proteins, slower progression through the cell cycle, reduction in protein synthesis, disruption of cellular cytoskeletal elements, and changes in membrane permeability. However, critical differences may also be observed (reviewed by Sonna et al., 2002).

Heat stress, either short-term or long-term, triggers the expression of HSP (reviewed by Hassan et al., 2019). HSPs promote survival by suppressing apoptosis, but there is a limit. Cells that have been exposed to severe heat stress are more susceptible to apoptosis than those subjected to mild heat stress. HSP70 can mediate the inhibition of heat-induced apoptosis through suppression of cytochrome c release, the inhibition of caspase activity, the inhibition of c-Jun N-terminal protein kinase (JNK) activation, the suppression of Apaf-1 oligomerization, and the inhibition of procaspase enrollment (reviewed by Hassan et al., 2019).

We expected a higher abundance of HSP70 in oocytes matured under thermal stress; however, in the present study, the abundance of this protein was higher in oocytes matured at 37 °C because it was significantly higher than in those matured at the control temperature, unlike the abundance at 40 °C, which was similar to 37 °C and 38.5 °C. Edwards and Hansen (1997) demonstrated that exposure of COCs to 41 °C for 12 h did not affect HSP70 protein abundance in oocytes. However, a direct comparison between both studies was not easy because of the different methods used. In addition to differences in the culture systems, different temperature regimens (41 °C/12 h vs. 40 °C/24 h) were used. It should also be noted that with *Bos taurus indicus,* we used animals specially adapted to high temperatures as oocyte donors. Therefore, the differences in the results can be explained clearly. However, the comparison also shows that, if at all, in contrast to cumulus cells, HSP70 in oocytes was only minimally affected by heat stress during IVM.

The abundance of HSP70 in cumulus cells increased, as expected, after heat stress. Khan et al. (2020) reported increased mRNA and protein expression of HSP70 after acute heat stress during the culture of bovine granulosa cells. The increase in apoptosis, decrease in cell viability, reduction of estradiol and progesterone secretion, and accumulation of intracellular reactive oxygen species (ROS), were also observed. The authors suggested that this platform was suitable for understanding the mechanism by which heat-stressed bovine granulosa cells could affect the quality of oocytes and developing embryos. This hypothesis is sensible as a previous study suggested the oocyte-protective effect of cumulus cells mediated by HSP70 (Edwards and Hansen, 1997).

Despite the expected reaction of cumulus cells after heat stress, in the present study, embryo development was impaired after chronic heat stress, probably because of the long exposure to stress conditions (24 h) that may overwhelm the protection capacity. In addition, Saadeldin et al. (2018) showed that camel oocytes were more sensitive to heat stress than cumulus cells. In response, cumulus cells responded, showing a higher abundance of HSP70 and HSP90 than oocytes and a similar relative abundance of apoptotic genes (BAX and P53) compared with the unstressed cumulus cells. In contrast, the expression of apoptosis-related genes was higher in stressed oocytes than in those matured at the control temperature.

After COC maturation at 37 °C, we observed the lowest amount of HSP70 in cumulus cells; the opposite was observed in oocytes. Many *in vitro* cultures of granulosa cells (GCs) are performed at 37 °C, which is not stressful for GC (Yenuganti and Vanselow, 2017; Baufeld et al., 2019). Cumulus cells are important for the thermal protection of oocytes, as they provide thermoprotective molecules, such as glutathione (Saadeldin et al., 2019) through gap junctions (Buccione et al., 1990). They also provide extracellular thermoprotectants or produce regulatory molecules that activate thermoprotective mechanisms within the oocyte (Edwards and Hansen, 1996). In addition, it was demonstrated that the effectiveness of gap junction-communication between cumulus cells and oocytes was reduced after heat stress (Campen et al., 2018). The disturbance of oocyte and embryo development could be due to the lack of HSP70 protection for cumulus cells at 37 °C, a stress temperature only for oocytes.

HSP90, which plays a key role in the maturation of many proteins and is important for several cellular processes, such as signaling, proteostasis, epigenetics, telomere maintenance, innate immunity, and others, interacts with at least 10% of all proteins produced by eukaryotic cells (Scheufler et al., 2000).

HSP90 was most abundant in cumulus cells and oocytes after IVM at 37 °C. Tang et al. (2016) observed an increase in *HSP90* gene expression in rat myocardial cells subjected to heat stress, followed by a decrease in its expression following an increase in the stress duration. In the present study, we only analyzed the HSPs after IVM for 24 h; therefore, we do not know if the behavior of the respective proteins changed during the maturation period. It could be that the high abundance of both HSPs during the whole maturation at 37 °C reflects a higher thermosensitivity of oocytes to cold stress. In contrast, another possibility was that the low amount of HSP90 observed after 24 h at 40 °C was a result of degradation during maturation due to chronic stress. New experiments showing the behavior of these proteins during different time points of maturation would be necessary to answer these questions properly.

It was demonstrated that high levels of HSP70 could sometimes block protein folding and that HSP90 efficiently buffered these detrimental effects, providing the cell with a robust folding machine (Morán-Luengo et al., 2018). These authors suggested a stop-start mechanism for the cooperation of the cascade in protein folding, allowing the protein to reenter a new folding cycle. Considering the very dynamic and complex interactions of HSP70 and HSP90, new experiments are necessary to clarify the behavior of these proteins during the process of chronic stress.

In summary, a stress of ±1.5 °C compared with the ideal oocyte maturation temperature had negative effects on the cleavage and blastocyst rates but did not affect the rate of PB extrusion in zebu cows.

The responses to cold and heat stress appear to be different. At 37 °C, the cumulus cells were probably not stressed and the efforts of the oocytes alone were not sufficient to prevent impaired embryo development. After chronic stress at 40 °C, only HSP70 was found extensively and exclusively in cumulus cells. Therefore, there may be a signal for resistance of cumulus cells and simultaneously a signal or the exhaustion of the HSP70-HSP90 system in oocytes. Further study is necessary to answer these questions.

## Conclusion

In conclusion, moderately high (40 °C) and low (37 °C) thermal stress applied for 24 h during IVM is detrimental to oocyte developmental competence and is accompanied by changes in the abundance of the HSP70 and HSP90 proteins, especially in cumulus cells.
